# Allergenicity of Alternative Proteins: Reduction Mechanisms and Processing Strategies

**DOI:** 10.1021/acs.jafc.5c00948

**Published:** 2025-03-19

**Authors:** Deniz Günal-Köroğlu, Gulsah Karabulut, Gulay Ozkan, Hilal Yılmaz, Büşra Gültekin-Subaşı, Esra Capanoglu

**Affiliations:** † Department of Food Engineering, Faculty of Chemical and Metallurgical Engineering, Istanbul Technical University, 34469 Maslak, Istanbul, Türkiye; ‡ Department of Food Engineering, Faculty of Engineering, Sakarya University, 54050 Sakarya, Türkiye; § Department of Biotechnology, Faculty of Science, Bartın University, 74100 Kutlubey Campus, Bartın, Türkiye; ∥ Center for Innovative Food (CiFOOD), Department of Food Science, Aarhus University, Agro Food Park 48, Aarhus N 8200, Denmark

**Keywords:** IgE, gut microbiota, immune responses, allergen reduction, processing methods

## Abstract

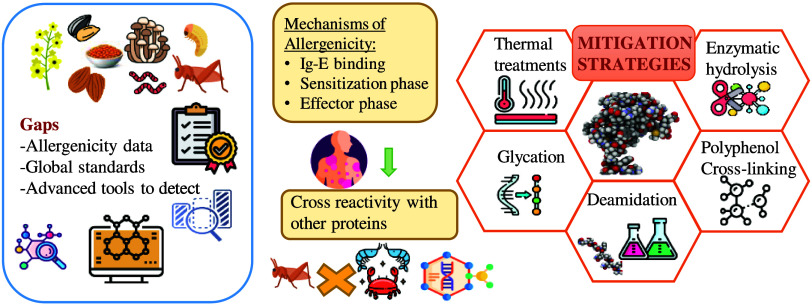

The increasing popularity of alternative proteins has raised concerns about allergenic potential, especially for plant-, insect-, fungal-, and algae-based proteins. Allergies arise when the immune system misidentifies proteins as harmful, triggering IgE-mediated reactions that range from mild to severe. Main factors influencing allergenicity include protein structure, cross-reactivity, processing methods, and gut microbiota. Disruptions in gut health or microbiota balance heighten risks. Common allergens in legumes, cereals, nuts, oilseeds, single-cell proteins, and insect-based proteins are particularly challenging, as they often remain stable and resistant to heat and digestion despite various processing techniques. Processing methods, such as roasting, enzymatic hydrolysis, and fermentation, show promise in reducing allergenicity by altering protein structures and breaking down epitopes that trigger immune responses. Future research should focus on optimizing these methods to ensure that they effectively reduce allergenic risks while maintaining the nutritional quality and safety of alternative protein products.

## 1 Introduction

The alternative protein sector has rapidly emerged as a growing industry, fueled by increasing demand for protein ingredients driven by population growth, evolving dietary preferences, and the need for sustainable food systems. The development of plant proteins requires advancements in extraction, functionalization, and addressing challenges related to allergenicity, flavor, and texture.^1^ While these proteins offer promising nutritional and functional properties, their introduction into diets may lead to adverse immune reactions in sensitive individuals. Understanding the allergenic potential of alternative proteins is thus crucial for ensuring consumer safety and building trust in this innovative food category.

An allergen is a substance, often a food protein, defined as an adverse reaction caused by antigen-specific immune mechanisms after exposure to a specific food in sensitized individuals.^2−4^ Cereals containing gluten (e.g., wheat, barley, rye), crustaceans, eggs, fish, milk, tree nuts (e.g., almonds, cashews, walnuts), peanuts, and soybeans are referred to as the “Big Eight” food allergens,^4^ which account for almost 90% of food allergies.^5^ The FASTER Act established sesame as the ninth major food allergen in the U.S.^6^ In the European Union, 14 allergens are recognized under EU law, including additional molluscs, celery, lupin, sesame, mustard, and sulphites.^7^ While current allergenicity risk assessments focus on known allergies and cross-reactivity, they inadequately predict the sensitization to novel or processed proteins.^8^ Cases like mealworm,^9^ cricket,^10^ algae,^11^ mycoprotein^12,13^-induced allergies and modified gluten^14^ reactions highlight this gap. Cross-reactivity between insect proteins with common allergens from crustaceans and house dust mites is well-established, largely due to shared allergenic proteins such as tropomyosin and arginine kinase.^15−18^ Similarly, algal proteins often contain epitopes that trigger IgE-mediated cross-reactivity in individuals allergic to crustaceans and fish.^19,20^ Additionally, Quorn patties^13^ have been linked to cross-reactions between fungal proteins and airborne mold allergens.^13^ Current allergenicity assessments on novel proteins primarily focus on known allergens and cross-reactivity, often overlooking the potential sensitization risks posed by novel or processed proteins. Since many novel proteins, such as those from insects, algae, and mycoproteins, share structural similarities with established allergens, they may induce IgE-mediated cross-reactive responses rather than being classified as primary allergens.

Food allergies result from immune responses to dietary antigens, affecting organs such as the skin and gastrointestinal tract, with symptoms ranging from mild to severe, including anaphylaxis.^2,21^ They are classified into IgE-mediated, non-IgE-mediated, and mixed reactions.^2,3,8^ Protein structure and amino acid sequence influence allergenicity, with denaturation reducing IgE binding.^22^ Detection methods include *in vivo* (e.g., Skin Prick Test, Double-Blind Placebo-Controlled Food Challenge) and *ex vivo/in vitro* assays like ELISA and ImmunoCAP ISAC.^3,21,23,24^ Early strategies such as breastfeeding and introducing allergen proteins promote oral tolerance.^25^ Nutritional interventions and treatments like specific immunotherapy and pharmacological options (e.g., adrenaline, antihistamines, biologics) offer relief, while emerging approaches like probiotics and fecal microbiota transplantation show potential.^3^

Different methods have been explored to reduce the allergenicity of alternative proteins, with promising results observed for certain approaches. Thermal processes, such as frying, boiling, steaming, microwave applications, and high-pressure thermal treatments, can denature proteins and reduce their IgE-binding capacity.^26−30^ Fermentation and enzymatic hydrolysis facilitate the breakdown or modification of proteins due to the breakdown of conformational and linear IgE-binding epitopes and the generation of small peptides and free amino acids.^26,31−36^ Furthermore, interactions with polyphenols can mask allergenic epitopes on proteins, reducing their recognition.^37−39^ Additionally, combined^40−43^ or novel^20,44−46^ applications have been highlighted as a highly effective strategy for reducing the allergenicity. The effectiveness of these methods varies depending on the food matrix and processing conditions, often requiring combined approaches for optimal allergen management. Nonetheless, it is important to note that while these methods show promise, validation through *in vivo* tests and clinical studies is essential to confirm their efficacy.

This review examines the allergenic reduction of alternative proteins, with a focus on legumes, cereals, nuts, oilseeds, insects, and single-cell proteins. It emphasizes the mechanisms by which these proteins trigger immune responses, particularly IgE-mediated reactions, and explores factors influencing their allergenicity, such as protein structure, genetic predisposition, gut microbiota, and immune regulation. The text details allergenic proteins, cross-reactivity between species, and food processing methods aimed at reducing allergenicity.

## 2 Allergic Response of Alternative Proteins in the Body

With the rising demand for alternative proteins driven by sustainability and dietary diversity, concerns about allergenicity have emerged. Unlike traditional protein sources, alternative proteins from plants, insects, fungi, and, macro- and microalgae are novel to many consumers and can elicit immune responses, posing unique food safety challenges. Understanding how these proteins trigger allergic reactions is essential for developing strategies to reduce allergenicity, allowing manufacturers to leverage their benefits while minimizing adverse effects.

Food allergies occur when the immune system mistakenly identifies certain food proteins as harmful, triggering an abnormal immune response. While the innate immune system acts as a general defense, adaptive immunity, involving antibody production, mediates these reactions. Even trace amounts of allergens can cause symptoms ranging from mild discomfort to severe anaphylaxis, including digestive issues, skin reactions, and respiratory inflammation.^47^ Unlike food intolerances, which arise from nonimmune factors like enzyme deficiencies, food allergies result from dysregulated immune responses.^48^

The food industry’s expansion into alternative protein sources, primarily derived from plants, seeds, legumes, and other novel ingredients, necessitates evaluating their allergenic potential. The rise of alternative protein sources, such as those from plants, seeds, legumes, and tubers, highlights the need to assess their allergenic potential. Many of these sources, like nuts and cereals, are known allergens contributing significantly to global food allergies.^49^ Structural similarities to existing allergens can cause cross-reactivity, triggering immune responses. Ingestion of these proteins may initiate immune cascades at the gastrointestinal epithelium. Additionally, processing or genetic modification can alter allergenic properties, increasing risks for consumers.^8^

When allergens enter the gastrointestinal tract, epithelial cells release pro-inflammatory cytokines like thymic stromal lymphopoietin (TSLP), activating dendritic cells. These cells process allergens and present them to T-helper cells, which stimulate B cells to produce allergen-specific IgE antibodies. This sensitization creates an immune memory, priming the system for faster and more severe reactions upon re-exposure.

IgE-binding activity is often assessed to predict sensitization, as most food allergies are IgE-mediated^50^ leading to immediate symptoms like urticaria, anaphylaxis, or oral allergy syndrome within 2 h of exposure.^2,8^ Specific forms, such as food-dependent exercise-induced anaphylaxis (FDEIA), also fall under this category and often result in acute, severe reactions.^2,8^ IgE-mediated allergies occur when the immune system loses tolerance to benign food antigens, producing specific IgE antibodies during initial sensitization. These antibodies bind to high-affinity receptors on mast cells and basophils, which release histamine and other mediators upon re-exposure, triggering allergic symptoms.^3^ Normally, oral tolerance suppresses IgE production,^51^ but disruptions in this process lead to sensitization of mast cells and basophils.^47^ IgE-mediated food allergies can cause immediate symptoms after consuming allergens like eggs, milk, wheat, crustaceans, or peanuts. Special forms include FDEIA, triggered by exercise within 2 h of eating certain foods, and oral allergy syndrome, a localized reaction to fresh produce linked to pollen allergies. Symptoms may affect the skin, respiratory or digestive systems, and circulation, with treatments like antihistamines and heat-treated foods often providing relief.^2^

**Figure 1:**
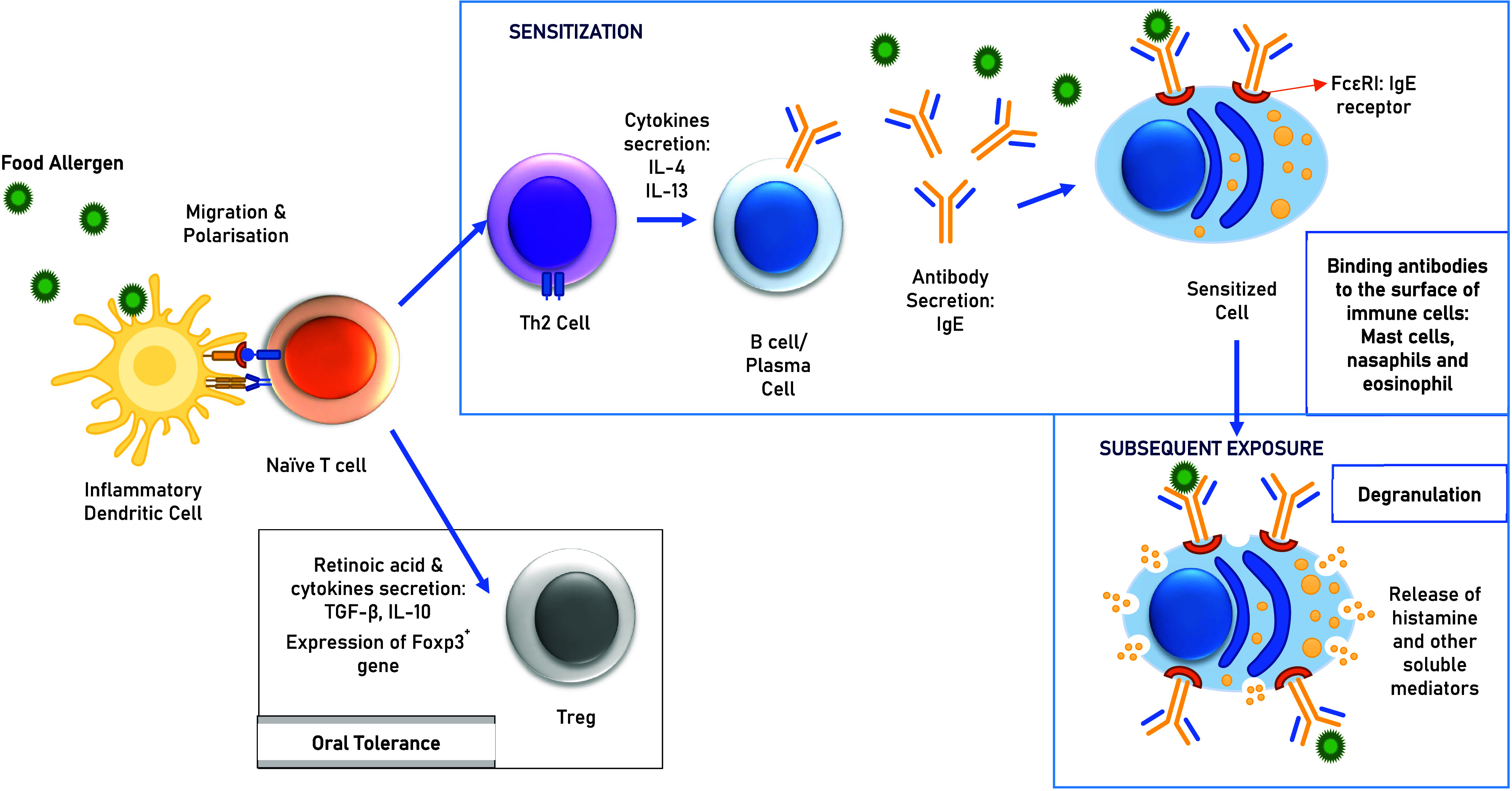
Mechanism of immune sensitization and allergic response with oral tolerance pathway.

IgE-mediated food allergies involve a type I hypersensitivity response,^52^ developing in two phases: sensitization and effector (Figure 1).^51^ Sensitization takes days to weeks, while the effector phase occurs within minutes of allergen re-exposure.

During sensitization, food antigens cross the epithelial barrier, triggering cytokine release (e.g., TSLP) and activating dendritic cells. These cells present antigens to naïve T cells, which differentiate into Th2 cells, releasing cytokines like IL-4 and IL-13 to stimulate B cells to produce IgE antibodies. IgE binds to mast cells and basophils, sensitizing them.^53^ Impaired regulatory T cells (Tregs) and certain T follicular helper cells (Tfh13) contribute to overactive immune responses, increasing IgE production and enhancing allergic reactions.^52^ In the effector phase (subsequent exposure), re-exposure to allergens cross-links IgE on sensitized mast cells and basophils, triggering degranulation and the release of mediators like histamines, leukotrienes, and prostaglandins.^47^ The early phase is driven by histamine and platelet-activating factor (PAF) and is regulated by T lymphocytes (Treg).^3^ Histamine increases vascular permeability, causing swelling and itching, while other mediators recruit eosinophils.^47^ The late phase involves cytokines such as IL-4, IL-5, and IL-13,^3^ leading to chronic inflammation and tissue damage, with symptoms ranging from hives to severe reactions like anaphylaxis.^47^ Understanding these mechanisms aids in developing diagnostic and therapeutic strategies.

Neonatal and infantile gastrointestinal allergy is a non-IgE-mediated food allergy that causes digestive issues, often triggered by cow’s milk, soy, or rice, and typically resolves by age two.^2,3,8^ These allergies involve T cells and affect the gastrointestinal tract, leading to conditions like eosinophilic esophagitis and celiac disease. Symptoms include chronic inflammation, often with eosinophils or neutrophils. Diagnosis lacks specific biomarkers, and the immune cells involved are not fully understood.^54^ The microbiota plays a key role in maintaining food allergy tolerance.^3^

Oral tolerance is the immune system’s ability to accept food proteins without triggering allergic reactions, beginning in the gut-associated lymphoid tissues (GALT), including Peyer’s patches and mesenteric lymph nodes (mLNs).^55^ Food antigens are processed in mLNs, where regulatory T cells (Tregs) are activated, promoting immune tolerance.^56,57^ CD103+ dendritic cells help convert naïve T cells into Tregs and induce IgA production via retinoic acid and TGF-β signaling.^21,57^ The gut microbiota and stromal cells in mLNs support Treg development, especially Foxp3^+^ Tregs, maintaining immune balance.^57^ The intestinal epithelial barrier, composed of the mucus layer, epithelial cells, and lamina propria, regulates nutrient processing and immune responses.^58^

**Figure 2:**
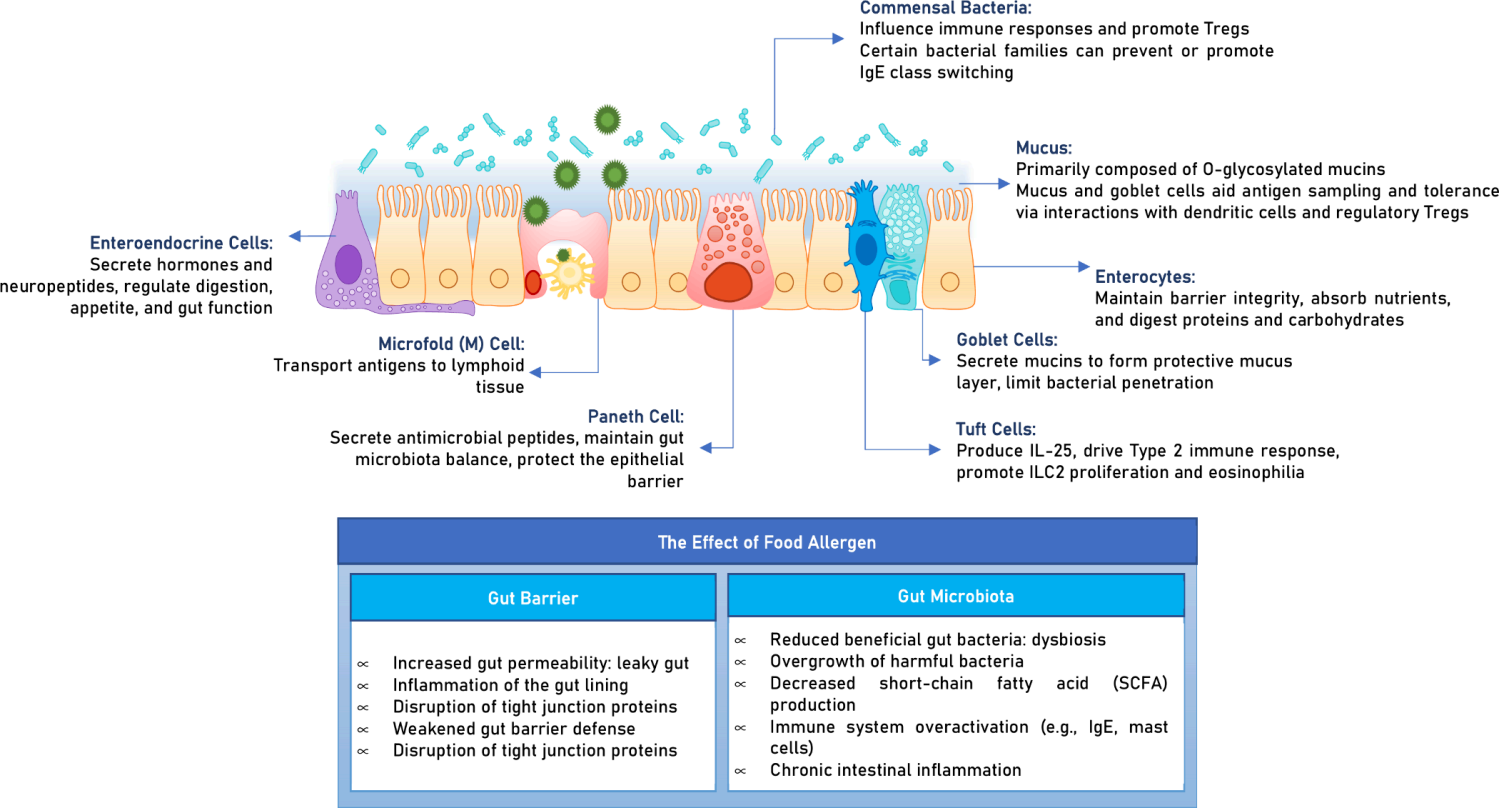
Impact of food allergens on the gut barrier and microbiota: cellular and molecular mechanisms.

Genetic factors, like the SERPINB10 gene, influence food allergy susceptibility by interacting with immune cells through antigen presentation and signaling. Specialized cells, including goblet, Paneth, tuft, and enteroendocrine cells, contribute to immune modulation and barrier protection. M cells, goblet cells, and dendritic cells transport antigens, while tight junctions maintain barrier integrity. Disruptions caused by diet, lifestyle, microbes, or inflammation can increase antigen exposure, leading to allergies.^59^ Increased intestinal permeability activates inflammatory pathways, including TLR4 and NF-κB, triggering cytokine release and overactivation of the Th2 pathway, promoting sensitization and allergic responses.^58^

Most allergen sensitization occurs in the small intestine, but the colon’s microbiota also influences this process.^59^ The gut microbiome regulates immune responses, mucosal barrier function, and Treg development, with commensal bacteria strengthening the intestinal barrier and promoting Treg differentiation to prevent food allergies.^60^ Children with food allergies have a microbiota characterized by bacteria linked to inflammation and a reduced abundance of immune-tolerance-promoting bacteria compared to healthy children.^61,62^ Specific bacterial strains like *Lactobacillus*, *Bifidobacterium*, and *Lachnospiraceae* produce short-chain fatty acids (SCFAs) (acetate, propionate, butyrate),^63^ which enhance gut barrier integrity, promote Treg expansion, and reduce IgE levels by modulating dendritic cells and B cells, leading to anti-inflammatory effects.^64,65^

### 2.1 Key Factors Contributing to Allergenic Reactions

The allergenicity of alternative proteins is influenced by their structural characteristics, particularly the presence of specific epitopes recognized by the immune system.^66^ Epitopes can be linear (continuous amino acid sequences) or conformational (dependent on protein folding), with linear epitopes retaining allergenicity even after processing, while conformational epitopes may lose it.^67,68^ Proteins with tightly packed structures, disulfide bonds, or glycosylation are more resistant to enzymatic breakdown, increasing allergenicity. Stability in the gastrointestinal tract also plays a role—proteins that resist digestion are more likely to trigger immune responses. Proteins with tightly packed structures, disulfide bonds, or glycosylation are more resistant to enzymatic breakdown, increasing allergenicity. Stability in the gastrointestinal tract also plays a role—proteins that resist digestion are more likely to trigger immune responses.^69^ For alternative proteins, complex or novel structures from plants, fungi, or insects may enhance resistance to digestive breakdown, and processing techniques can create new allergenic epitopes.^68^ Understanding these structural properties is crucial to predicting and mitigating allergenic risks.^66^

Genetic predisposition plays a key role in determining susceptibility to allergic reactions, including those from alternative proteins. Polymorphisms in genes such as interleukin-4 (IL-4) and interleukin-13 (IL-13) regulate T-helper type 2 (Th2) immune responses, a hallmark of allergies.^23^ Variants in human leukocyte antigen genes influence antigen presentation to T-cells, shaping immune reactivity.^70^ The FCER1 gene, encoding the high-affinity receptor for IgE on mast cells and basophils, can amplify allergic responses when mutated.^53^ Additionally, mutations in filaggrin, a gene involved in epithelial barrier function, can enhance allergen penetration and sensitization.^71^ Together, these genetic factors underscore the interplay between inherited traits and immune hyperreactivity, increasing vulnerability to allergens in alternative proteins.^72^

Environmental factors also significantly influence the development and severity of allergic responses. The gut microbiota plays a significant role in modulating immune responses; dysbiosis, or an imbalance in microbial populations, has been linked to increased allergic sensitization.^64^ In addition to exposure to pollutants or other lifestyle factors, such as stress, smoking, and sedentary behavior, dietary patterns during early life or adulthood also impact immune response. For example, diets high in ultraprocessed foods and low in fiber have been linked to dysbiosis of the gut microbiome, which plays a critical role in immune regulation. Conversely, exposure to diverse dietary proteins early in life may reduce allergenic risk through oral tolerance mechanisms.^73^

Additionally, factors like urbanization, which limit exposure to diverse microbial environments, are associated with higher allergy prevalence, supporting the “hygiene hypothesis” that reduced microbial exposure leads to an overactive immune response.^74^ Recognizing these environmental contributors underscores the importance of dietary and lifestyle interventions in reducing allergy prevalence.

As novel protein sources including legumes, insects, fungi, and lab-grown proteins gain popularity, concerns about their allergenic potential and cross-reactivity have increased. These proteins often resemble traditional allergens structurally, leading to cross-reactivity.^75^ For example, insect proteins like tropomyosin share similarities with shellfish allergens, which may trigger reactions in shellfish-allergic individuals.^15^ Plant proteins, such as those from chickpeas and lentils, exhibit homologous epitopes with peanuts, increasing cross-reactivity risks.^76^ Fungal proteins may also contain stable epitopes resistant to digestion, heightening their potential for IgE-mediated responses.^77^ Food processing can further impact allergenicity by modifying protein structures or exposing hidden epitopes, potentially enhancing or reducing allergenic risks.^78^ Additionally, alternative proteins might cross-react with airborne allergens, complicating their allergenic profiles. To address these concerns, careful evaluation of novel protein sources through epitope mapping, allergenicity testing, enzymatic hydrolysis, and genetic modifications is crucial to minimize risks and ensure consumer safety.

### 2.2 Advanced Technologies for Allergen Detection

Identifying allergens in food proteins using classical methods relies on immunological, clinical, and physicochemical methods to evaluate IgE reactivity, immune response activation, and protein stability.^31^*In vitro* immunological assays, such as ELISA, Western blotting, the Basophil Activation Test (BAT), and the Radioallergosorbent Test (RAST), are commonly used to detect IgE binding and immune system recognition of specific proteins.^40^ These methods help determine whether a protein has the potential to trigger allergic reactions by examining interactions with antibodies from allergic individuals.

*In vivo* methods, including the Skin Prick Test (SPT), Intradermal Test, and Oral Food Challenge (OFC), provide direct clinical evidence of allergenicity by measuring immediate allergic responses, such as wheal and flare reactions or systemic symptoms after controlled exposure.^43^^42^ Additionally, digestibility and stability assessments play a critical role in allergen identification. Simulated Gastric Fluid (SGF) and Simulated Intestinal Fluid (SIF) tests evaluate whether a protein is resistant to digestion, as more stable proteins tend to be more allergenic.^79^ However, while classical methods provide important allergenicity insights, they have certain limitations, including low throughput, time-consuming protocols, and the inability to fully characterize allergenic determinants at a molecular level. To overcome these challenges, omics technologies and bioinformatics approaches are increasingly used to complement traditional allergen assessment.

The application of omic technologies in allergen detection represents a transformative approach to understanding and mitigating allergenic risks associated with proteins. These technologies integrate computational tools, structural analyses, and chemical modification strategies to identify and disrupt allergenic epitopes effectively.^80^ While they offer unprecedented precision in predicting and modifying allergenic proteins, their current limitations lie in translating computational and in vitro findings into in vivo outcomes. Allergenicity assessments often require validation through human subject studies, which are not only resource-intensive but also ethically and logistically complex.^81^ Moreover, cross-reactivity studies—critical for ensuring the safety of alternative proteins—remain underexplored in many cases. Addressing these gaps is crucial for advancing the reliability and applicability of omic technologies in allergen detection.

Bioinformatics tools such as AllerTOP, AlgPred, and AllergenPro predict potential IgE-binding epitopes by analyzing sequence motifs and structural characteristics.^82^ These predictions focus on regions prone to immune recognition, including proline- and lysine-rich segments. Such regions are prime targets for chemical modification, including glycation or conjugation with polyphenols, to disrupt or mask allergenic epitopes.^83^ This targeted approach allows for efficient interventions that minimize allergenic potential.

Molecular docking and dynamics simulations are employed to model interactions between allergenic proteins and IgE antibodies.^84^ These techniques provide detailed insights into the binding affinity and structural changes induced by chemical treatments such as enzymatic hydrolysis or polyphenol conjugation. For example, simulations can predict how specific chemical agents, like epigallocatechin gallate (EGCG), disrupt critical IgE-binding interactions by forming covalent or noncovalent bonds with allergenic proteins.^85^

Sequence alignment tools, including BLAST and FASTA, identify homologous regions between novel proteins and known allergens. Cross-reactivity risks are assessed by comparing conserved sequence motifs or structural domains.^86^ This analysis supports the design of targeted chemical modifications to disrupt homologous epitopes, thereby reducing the likelihood of immune cross-reactivity while maintaining protein functionality.

Programs such as PyMOL, Chimera, and AlphaFold generate high-resolution three-dimensional models of allergenic proteins. These models visualize the spatial arrangement of epitopes, identifying regions suitable for enzymatic or chemical modification.^87^ This structural information guides experimental approaches, such as introducing site-specific mutations or targeted chemical reactions, to alter allergenic epitopes effectively.

Computational modeling of chemical interactions, such as glycation, oxidation, or polyphenol binding, predicts their effects on allergenic epitopes. For instance, EGCG and other phenolic compounds can interact with allergenic proteins to reduce IgE recognition by masking or modifying critical binding sites.^38,39^ These simulations enable the rational design of treatment processes, minimizing allergenic potential while preserving the protein’s structural integrity.

Simulations of processing methods, such as enzymatic hydrolysis combined with thermal or nonthermal treatments (e.g., pulsed electric fields or plasma treatment), evaluate their impact on protein structure and allergenicity. These computational models help optimize process parameters to maximize epitope disruption while maintaining protein functionality.^88^ For example, combining mild thermal treatments with electric field has been shown to enhance the reduction of allergenic potential compared to either method alone.^89^

The integration of bioinformatics and chemical analysis enables precise interventions to reduce allergenic potential in proteins. Techniques such as glycation, enzymatic hydrolysis, and polyphenol conjugation effectively mask or alter allergenic epitopes, while bioinformatics tools predict and validate these modifications’ effects on IgE binding. Computational models streamline the design and optimization of allergen mitigation strategies, bridging the gap between experimental and theoretical approaches.

This combined methodology sets the stage for innovative solutions in allergen reduction, advancing the development of safer, hypoallergenic alternative proteins. These approaches hold promise for broadening the acceptance and application of alternative protein sources in food, pharmaceuticals, and functional products, ultimately improving consumer safety and expanding market potential. Future research should focus on integrating machine learning algorithms for improved epitope prediction, exploring novel chemical treatments, and validating these findings through in vivo and clinical studies to ensure efficacy and safety.

## 3 Allergenicity and Mitigating in Alternative Proteins

Table 1 shows the allergen proteins for alternative sources, including the WHO/IUIS Allergen Database and literature. According to European Union Annex II of Regulation EC no 1169/2011,^7^ 14 known food groups are listed, frequently consumed foods are missing from this list.

**Table 1:** Comprehensive Overview of Food Proteins and Allergens: Sources, Molecular Characteristics, and IUIS Classifications^a^

	**Proteins**	**Allergens**	**IUIS names**	**MW (kDa)**	**Refs**
**Legumes**	White bean (*Phaseolus vulgaris*)	nsLTP1^b^	Pha v 3^b^	8.8–9	(116)
	Mung bean (*Vigna radiata* L.)	PR-10	Vig r 1	16	(104), (116)
		8S Globulin (vicilin)^b^	Vig r 2^b^	52	
		Seed albumin^b^	Vig r 4^b^	30	
		CSBP (Bet v 1)	Vig r 6	18	
	Lupin (*Lupinus angustifolius*)	7S Globulin (vicilin)^b^ nsLTP^b^	Lup an 1^b^	55–61	(116), (201)
			Lup an 3^b^	11	
	Lupin (*Lupinus albus*)	Profilin^b^	Lup an 5^b^	15	
	Chickpea (*Cicer arietinum*)	Late embryogenesis protein	Cic a 1^b^	42	(116)
	Peas (*Pisum sativum* L.)	Vicilin^b^	Pis s 1^b^	44	(116), (202)
		Convicilin^b^	Pis s 2^b^	63	
		nsLTP	Pis s 3	9.5	
	White bean (*Phaseolus vulgaris*)	nsLTP1^b^	Pha v 3^b^	8.8–9	(116)
	Soybean (*Glycine max*)	Hydrophobic protein	Gly m 1	7	(76), (116)
		Defensin	Gly m 2	8	
		Profilin	Gly m 3	14	
		PR-10^b^	Gly m 4^b^	17	
		7S Globulin (vicilin)^b^	Gly m 5^b^	subunits	
		11S Globulin (legumin)^b^	Gly m 6	subunits	
		SBP	Gly m 7	76.2	
		2S Albumin	Gly m 8	28	
**Cereals**	Wheat (*Triticum aestivum*)	Ω-5 Gliadin^b^	Tri a 19	65	(116), (117)
		γ-Gliadin	Tri a 20	35–38	
		High molecular weight glutenin^b^	Tri a 26	88	
		Low molecular weight glutenin^b^	Tri a 36	40	
		Amylase inhibitors^b^	Tri a 15, 28-30, 40	13–16	
	Rice (*Oryza sativa*, ssp. *japonica*)	Beta-expasin	Ory s 1	35	(203)
		Profilin A	Ory s 12	14	
	Rye *(Secale cereale)*	Gamma-secalin^b^	Sec c 20	70	(204)
	Barley *(Hordeum vulgare)*	Profilin	Hor v 12	14	(136)
		α-amylase inhibitor precursor	Hor v 15	14.5	
		β-amylase	Hor v 17		
		γ-hordein^b^	Hor v 20	34	
	Maize/Corn *(Zea mays)*	α-amylase inhibitor^b^		14	(139)
		LTP^b^	Zea m 14	9	
		endochitinases		30	
		zein-α precursor		26	
		zein-β precursor		19	
**Nuts**	Cashew (*Anacardium occidentale*)	7S Vicilin^b^	Ana o 1^b^	50	(142), (143)
		11 S Legumin^b^	Ana o 2^b^	33	
		2S Albumin^b^	Ana o 3^b^	13	
	Hazelnut (*Corylus avellana*)	Bet v1 homologue	Cor a 1	17	(116), (205)
		Profilin^b^	Cor a 2^b^	14	
		LTP^b^	Cor a 8^b^	9	
		11S legumin^b^	Cor a 9^b^	40	
		7S vicilin^b^	Cor a 11^b^	48	
		Oleosin	Cor a 12	17	
		Oleosin	Cor a 13	14–16	
		2S albumin	Cor a 14	10	
		Oleosin	Cor a 15	17	
		7S globulin seed storage protein (vicilin) containing N-terminal alpha-hairpinin (vicilin_N) peptides	Cor a 16	6–8 and 47.5	
	Peanut (*Arachis hypogaea* L.)	7S Vicilin^b^	Ara h 1^b^	64	(116), (206)
		2S Albumin^b^	Ara h 2^b^	17	
		11S Legumin^b^	Ara h 3^b^	60, 37	
		11S Legumin	Ara h 4	15	
		Profilin	Ara h 5	15	
		2S Albumin^b^	Ara h 6^b^	15	
		2S Albumin	Ara h 7	17	
		PR-10	Ara h 8	9.8	
		nsLTP1	Ara h 9	16	
		Oleosin	Ara h 10	14	
		Oleosin	Ara h 11	8, 12, 5.184	
		Defensin	Ara h 12	8, 11, 5.472	
		Defensin	Ara h 13	17.5	
		Oleosin	Ara h 14	17	
		Oleosin	Ara h 15	8.5	
		nsLTP2	Ara h 16	11	
		nsLTP1	Ara h 17	21	
		Cyclophilin	Ara h 18		
	Pictachio (*Pistacia vera*)	2S Albumin^b^	Pis v 1^b^	7	(149), (150)
		11S Globulin^b^	Pis v 2^b^	32	
		7S Vicillin	Pis v 3	55	
		Superoxide Dismutase	Pis v 4	25.7	
		11S Globulin^b^	Pis v 5^b^	36	
	Walnut (*Juglans regia* L.)	2S Albumin^b^	Jug r 1^b^	15–16	(116)
		7S vicilin	Jug r 2	44	
		nsLTP1	Jug r 3	9	
		11S globulin	Jug r 4	58.1	
		PR-10	Jug r 5	20	
		7S globulin	Jug r 6	47	
		Profilin	Jug r 7	13	
		nsLTP2	Jug r 8	9	
**Oilseeds**	Sunflower (*Helianthus annuus*)	2S albumin^b^	Hel a 6^b^	12–15	(155), (207), (208)
		Aeroallergen	Hel a 1	34	
		Profilin	Hel a 2	14.7	
		LTP	Hel a 3	9	
		Oleosins	NC	21–23	
	Rapeseed (*Brassica napus*)	Cruciferin (11S globulin)	NC	300–350	(36), (116), (159), (209)
		Napin (2S albumin)^b^	Bra o 3^b^	12–16	
		LTP	Bra o 2	3–9	
		2S albumin seed storage protein	Bra n 1	15	
	Sesame (*Sesamum indicum*)	2S Albumins^b^	Ses i 1^b^	9, 7	(160)
			Ses i 2^b^	45	
		Vicilins (7S Globulins)^b^	Ses i 3^b^	17, 15	
			Ses i 4^b^	52–5	
		Oleosins^b^	Ses i 5^b^	14	
			Ses i 6		
		11S Globulins Profilins	Ses i 7		
			Ses i 8		
	Hemp seed (*Cannabis sativa*)	2S Albumins^b^	NC	Variable (small, stable seed proteins)	(168), (208)
		7S Vicilins		45–50	
		11S Legumins		300–350 (hexamers)	
		Profilins		14	
**Insects**	Brown garden snail (*Helix aspersa*)	Tropomyosin	Hel as 1	36 kDa	(116)
	Silk moth (*Bombyx mori*)	Arginine kinase	Bomb m 1	42	(116)
		Tropomyosin	Bomb m 3	38	
		30 kDa hemolypmh lipoprotein PBMHP-6	Bomb m 4	30	
		30 kDa Lipoprotein 6	Bomb m 5	29	
		Hemolymph lipoprotein 3	Bomb m 6	28	
**Micro- and macro-algae**	Spirulina (*Arthrospira platensis*)	C-Phycocyanin (C-PC)^b^	NC	40–50	(210)
		Allophycocyanin		38	
		Phycobiliproteins		15–20	
	*Arthrospira maxima*	Spi mx	NC	NC	Yu, Li and Cen (2002)
		Beta- Phycocyanin			
	Chlorella spp.	Chl s	NC	13, 17, 19, 25–26, 46–50, 72	(184); Tiberg and Einarsson (1989)
	*Laminaria digitata*	Lam d	NC	NC	Kim et al. (2003); Sierra et al. (2015)
	Red Algae (*Rhodophyta*)	Phycoerythrin^b^	NC	Complex: 240; subunits: 20–25	(210)
		Mycosporine-like amino acids (MAAs) binding proteins^b^		12–14	
	Green Algae (*Chlorophyta*)	Cell wall proteins (Hydroxyproline-rich Glycoproteins)^b^	NC	30–70	(210)
	Macroalgae (Seaweed)	Porphyra-334 (from Nori)^b^	NC	10–15	(20)
		Fucoxanthin-associated proteins^b^		40–50	
	Mycoproteins (*Fusarium venenatum*)	Sourced by additives used during processing			(198)

a MW: Molecular weight, IUIS: International Union of Immunological Societies, LTP: Lipid Transfer Protein, NC: not classified.

b Major allergens.

The mitigation of allergenicity in alternative proteins is a rapidly evolving area of food chemistry and biochemistry. Foods undergo diverse processing methods prior to consumption to enhance functional, nutritional, and sensory attributes, as well as for preservation and detoxification. Common processing techniques include thermal treatment, high pressure, radiation, high-intensity ultrasound, and biochemical approaches. These methods can induce structural changes in food proteins, such as unfolding, aggregation, cross-linking, oxidation, and glycosylation, which directly influence allergenicity by disrupting conformational or linear epitopes.^90^ Additionally, processing-induced physicochemical modifications may affect the gastrointestinal digestibility, absorption kinetics through the mucosa, and antigen presentation to the immune system, thereby altering the allergenic response.^91^

The inherent biochemical characteristics of food allergens significantly contribute to their allergenicity. For example, the peanut allergen Ara h 1 demonstrates exceptional stability, resisting heat and digestive enzyme degradation. It also contains a glycan adduct that acts as a TH2 (T helper cell) adjuvant, enhancing its immunogenic potential.^92^

Chemical and biochemical approaches offer precise methods to mitigate allergenicity by modifying or breaking down allergenic proteins into less immunogenic fragments. Key strategies include glycation,^93^ and Maillard reactions,^94^ enzymatic hydrolysis,^27,28,32,40^ cross-linking with polyphenols,^38,39^ and deamidation.^95^

Glycation and Maillard reactions are among the most widely studied chemical processes for allergen reduction. Glycation involves the nonenzymatic attachment of reducing sugars to free amino groups in proteins, leading to structural changes that can mask allergenic epitopes and reduce IgE binding.^96^ Maillard reactions, occurring during heat processing, similarly alter protein structures through the formation of advanced glycation end-products (AGEs). These modifications can inhibit IgE recognition, effectively lowering allergenicity without severely impacting the protein’s nutritional quality.^94^

Enzymatic hydrolysis is a highly targeted approach that utilizes proteolytic enzymes to cleave allergenic proteins into smaller peptides and free amino acids.^97^ This process disrupts both conformational and linear epitopes, diminishing IgE-binding capacity. Enzymes such as papain, alcalase, and flavorzyme have been applied to legume proteins (e.g., mung beans and chickpeas), resulting in a notable reduction in allergenicity.^32,98^ The degree of hydrolysis is a critical factor, with extensive hydrolysis typically yielding smaller, less immunogenic peptides. However, partial hydrolysis may unmask hidden epitopes, necessitating careful control of hydrolysis conditions to achieve optimal allergen reduction.

Polyphenol-induced cross-linking and aggregation represent another effective biochemical strategy for allergen mitigation. Polyphenols, including chlorogenic acid, catechin, and tannins, can interact with allergenic proteins to induce structural changes that reduce their IgE-binding capacity.^95^ Cross-linking can lead to the formation of protein aggregates, effectively burying allergenic epitopes and rendering them less accessible to the immune system.^99^

Denaturation and deamidation alter the structural integrity of allergenic proteins, often rendering them less immunogenic. Deamidation involves the conversion of glutamine and asparagine residues into glutamic and aspartic acids, respectively.^100^ This process introduces negative charges that disrupt protein folding and reduce IgE binding. Deamidation has been successfully applied to gluten and soy proteins, decreasing their allergenicity while maintaining essential functional properties. Heat, mild acid, and alkali treatments facilitate deamidation, with the extent of modification depending on processing conditions.^95^

### 3.1 Legumes

Legumes are valuable plant-based protein sources due to their protein content, slow-digesting starches, dietary fibers, and low fat.^101^ However, they pose allergenicity risks, as IgE-binding proteins have been detected in many legumes.^51^ Allergens in lupin, soybean, and peanut must be labeled, while peas, beans, lentils, and chickpeas, even though they are not major allergens,^7^ may still contain IgE-binding proteins and exhibit allergenic potential.^102^ Legume allergens fall into three groups: storage proteins (cupin and prolamin superfamilies), profilins, and pathogenesis-related proteins (PR-10).^102,103^

The mung bean (*Vigna radiata* L.) contains allergenic storage proteins like globulins, albumins, and legumins.^104^ While its nutritional properties are well-studied, its allergenicity is less explored.^105^ Recently, Calcinai et al.^31^ investigated enzymatic hydrolysis via papain, alcalase and flavorzyme of mung bean proteins with papain, alcalase, and flavorzyme reduced allergenicity by breaking down IgE-binding epitopes and generating peptides and free amino acids.

Calcinai et al.^32^ studied on reducing the allergenicity of white bean byproducts through enzymatic hydrolysis via papain and alcalase found that all patients showed immunoreactivity to hydrolysates produced with papain or alcalase, likely due to antinutritional factors hindering protein digestion.^32^ Similarly, chickpea allergenicity after enzymatic hydrolysis. Papain converted residual proteins into free amino acids and peptides, with ∼40% of patients showing immunoreactivity, while alcalase hydrolysates showed no immunoreactivity. The findings suggest enzymatic hydrolysis cannot completely eliminate immunoreactivity and may even increase it for some allergens, such as 2S albumin, by exposing new IgE-binding epitopes.^32^

Among over 450 lupin species, those with lower quinolizidine alkaloid content, such as white (*L. albus*), yellow (*L. luteus*), blue or narrow-leafed lupin (*L. angustifolius* L.), and pearl or Andean lupin (*L. mutabilis* L.), are used in food formulations.^104^ Lupins are valued for their high protein (up to 44%), dietary fiber, and essential amino acids, making them suitable as flour, protein isolates, or concentrates for food fortification.^106^ However, lupins can cause allergic reactions, either through primary sensitization or cross-allergy with other legumes (e.g., soybean, peanut, lentils, chickpeas) due to high sequence similarity.^107^ Allergenic potential may be reduced by modifying allergen epitopes via food processing methods like fermentation via *Rhizopus oligosporus*,^33^ germination at 25 °C for 9 days,^108^ or roasting at surface temperatures of 98–242 °C,^109^ though *in vivo* and clinical validation is needed.

Peas (*Pisum sativum* L.) are valued for their nutritional, sustainable, and economic benefits.^110^ Pea proteins, similar to lupin allergens, share epitopes with other legume allergens, leading to serological cross-allergy.^111^ Efforts to reduce pea protein allergenicity found that lactic acid fermentation (*Lactobacillus plantarum*) followed by enzymatic hydrolysis (papain, Esperase, trypsin) achieved the greatest reduction in immunogenicity compared to other methods.^40^ However, these results have not been confirmed by *in vivo* or clinical studies yet.

Soybean proteins can cause severe allergic reactions like urticaria, rhinitis, swelling, anaphylactic shock, and death.^41^ Up to the present, various food technologies have been applied to reduce the allergenicity of soybean proteins.^112−114^ Among others, thermal treatments of boiling and autoclaving could be indicated as promising methods to decrease the allergenicity of soybean, suggesting degradation of proteins and structural changes depending on the time and temperature of the applications.^112^ For instance, the impacts of the effects of industrialized sterilization processes of boiling and autoclaving on the immunoreactivity of soybean protein were assessed by Pi et al.^29^ Allergenicity reductions of 43%–59% with boiling at 100 °C and 82%–83% with autoclaving at 121 °C for 20 min, highlighting autoclaving as more effective due to epitope destruction and structural changes.^29^

### 3.2 Cereals

Cereal proteins, classified by solubility, include water-soluble albumins, saline-soluble globulins, and alcohol-soluble prolamins (gliadin and glutenin), which are potential allergens.^115^ Allergenic cereals like wheat, rice, oat, maize, barley, sorghum, and millet exhibit varying allergenic proteins. Twenty-eight wheat allergens, reaching 35 when including other wheat types.^116^ Among them, α-amylase inhibitors allergens include α-amylase inhibitors, which are linked to baker’s asthma and wheat hypersensitivity, with proteins ranging from 12 to 98 kDa binding IgE in affected individuals. Prolamins, particularly ω-5 gliadin, are major triggers in wheat-dependent exercise-induced anaphylaxis and immediate wheat allergies, with strong IgE responses observed in children. Nonprolamins, glycoproteins, and profilins also contribute to wheat allergies, playing roles in enzymatic functions, immune responses, and cross-reactivity with pollen allergens.^117^

Processing the wheat-containing food biomass might modify the potential allergenicity of the wheat, which is also acknowledged as a mitigation strategy for allergen fractions. Particularly, thermal processing, fermentation, and enzyme/acid hydrolysis are the major treatments that are shown to have effects on wheat allergens in the literature. The most targeted allergen factors, to mitigate by varying processing approaches are α-amylase inhibitors/amylase trypsin inhibitors^118^ and gluten subunits gliadin and glutenin^119^ due to their severity and high probability of occurrence. However, processing methods and food matrices can sometimes create mega allergens in a way that increases their allergenic potential rather than reducing it.^120^ For example, the Maillard reactions, a form of food glycation, can alter the immunoreactivity of proteins by generating new epitopes or modifying existing ones. Depending on factors such as protein and sugar sources, processing conditions, and glycation duration, it may decrease, remain unchanged, or even increase allergenicity.^121^ Studies as reviewed by Rao et al.^121^ showed that wet-heated glycation generally reduces immunoreactivity more than dry-heated glycation, though processing differences may influence outcomes. Simonato et al.^122^ demonstrated that high-temperature baking can lead to the formation of highly IgE-reactive allergenic protein aggregates, particularly in the bread crust, likely due to Maillard-like reactions, whereas the allergenicity of the bread crumb was reduced. Novel approaches, including nonthermal treatments and biological methods, have shown potential for reducing wheat allergenicity in animal models, but further *in vivo* studies are needed.^45,119,123^

Phenolic interactions with wheat allergen proteins offer a potential alternative processing method. Cranberry extract was found to be the most effective polyphenol source among artichoke leaves, cranberries, apples, and green tea leaves for reducing IgE recognition of wheat gliadin in mice, which inhibited cellular degranulation and masked epitopes for IgE or IgG antibodies in wheat allergy patients.^124^ Complexation of wheat gliadin with chlorogenic acid or luteolin reduced IgG binding, with luteolin showing a stronger effect due to more covalent interaction sites.^125^ Additionally, removing free and bound phenolics from wheat protein fractions, particularly prolamins, lowered allergenicity.^126^ He et al.^127^ have shown that both noncovalent and covalent complexes of wheat gluten hydrolysate-chlorogenic acid can significantly reduce gluten allergenicity

Rice (*Oryza sativa*, ssp.) is rarely allergenic, with only a few cases of anaphylaxis reported in Asia, mainly caused by the lipid transfer protein cross-reacting allergen β-expansin (35 kDa)^115^ and Profilin A (14 kDa),^116^ which are airborne allergens. Rice allergy is often linked to non-IgE-mediated food allergies, like food-protein-induced enterocolitis syndrome, which may be an under-recognized risk.^128^ However, there is limited research on mitigating rice allergens through food processing. One study suggested that cooking methods like boiling, steaming, or microwaving may reduce potential rice allergenicity.^129^

The main allergenic compound in rye (*Secale cereale*) is γ-secalin (70 kDa), a gluten protein.^116^ Conventional ELISA-based methods are unsuitable for accurately determining rye prolamins and their allergenic risk.^130^ Since rye contains more than 50% γ-secalin, it cannot be considered gluten-free.^131^ A study using sourdough fermentation showed that while it altered protein size distribution and reduced secalin proteins, the allergenicity potential of rye protein increased.^132^ This highlights the need for specialized treatments to mitigate the allergenic risks of rye gluten protein.

Barley (*Hordeum vulgare*) contains the allergenic protein hordein, similar to secalin in rye and avenin in oats, and it may cross-react with wheat gluten (ω-5 gliadin).^133^ Barley has three more allergen-attributed compounds besides γ-hordein, the major allergen, which are profilin, α-amylase inhibitor precursor, and β-amylase.^116^ The beer industry has explored reducing allergenicity in barley, with treatments like *Trichoderma reesei*-expressed prolyl endopeptidase enzyme showing promise in lowering gluten content.^134^ An EFSA study investigated the safety of spent barley protein utilization for further novel food applications in terms of remaining allergenicity potential and indicated that it might still possess allergen factors.^135^ On the other hand, apart from food processing techniques, novel breeding strategies could be superior to producing ultralow gluten barley grains suitable for celiac and gluten-intolerant individuals’ consumption.^136^

Oats (*Avena sativa*) and maize (corn) are not listed with known allergens,^116^ and no oat-induced allergenicity cases have been reported in recent decades, with the exception of a rare anaphylactic reaction in a 7-year-old in 2013 linked to oat 12S seed storage protein.^137^ Even though oats have gluten-allergen genes, the expression level is not severe enough to make oats a gluten-allergen cereal.^138^ Furthermore, one of the most relevant and up-to-date maize food allergy studies was published in 2009, and LTP was demonstrated as the most significant maize allergen that might cause anaphylaxis in Swiss/Italian people.^139^ The literature does not indicate a specific food processing study aiming to reduce maize allergenicity. The only relevant study using extrusion for allergen reduction for soybean protein and flour mixes with cornmeal might be considered, which pointed out that with low moisture extrusion, it is possible to reduce the overall allergenicity (based on IgE immunoreactivity assay) by up to 86% with 20% moisture and 200 rpm screw speed.^140^

### 3.3 Nuts

Cashew nuts (*Anacardium occidentale*) can trigger allergic reactions, with key allergens including Ana o 1 (50 kDa vicilin-like protein), Ana o 2 (33 kDa legume-like protein), and Ana o 3 (13 kDa 2S albumin).^141−143^ Ana o 1 resists heat and proteolysis, while Ana o 3 is linked to severe reactions and is used as a clinical allergy predictor.^144,145^ Various thermal (boiling at 100 °C for 60 min, autoclaving at 138 °C/256 kPa for 30 min, pressured heating at 170 °C/7 bar for 120 s)^43^ and nonthermal methods (irradiation at 1–10 kGy)^44^ have been applied to reduce cashew nut allergenicity. Thermal treatments reduced skin prick test results significantly, with the greatest reduction achieved by combining pressured heating and amano enzyme treatment. Electrophoresis showed complete elimination of protein bands after autoclaving and pressured heating, and IgE binding was minimal with combined treatments.^43^ Irradiation at 1–10 kGy also reduced IgE binding to Ana o 3 and cytokine levels (IL-6, TNF-α) and histamine contents, particularly after 10 kGy.^44^

Hazelnut (*Corylus avellana*) is one of the tree nuts responsible for allergic reactions. Various heat treatments, including boiling, autoclaving, and pressured heating,^43^ were studied for their effects on hazelnut allergenicity. Results showed significant reduction in skin prick test responses for treated hazelnuts, with the most notable decrease after combined pressured heating and enzyme treatment. Electrophoresis and IgE binding further confirmed protein denaturation.^43^ Additionally, autoclaving, combined with dehydration or drying, also reduced allergenicity, as evidenced by a decrease in wheal size on skin and disappearance of specific protein bands in the region of 30–50 kDa and 10–22 kDa.^42^

Peanuts (*Arachis hypogaea*) are a major food allergen, with Ara h 1, Ara h 2, Ara h 3, and Ara h 6 being the most sensitizing proteins.^146^ To reduce allergenicity, various methods have been used, including roasting at 170 °C,^147,148^ thermal treatment (boiling at 100 °C for 60 min and autoclaving at 138 °C/256 kPa for 30 min), thermal treatment combined with pressure application (at 170 °C/7 bar for 120 s), enzymatic digestion (amano enzyme),^43^ as well as conjugation with phenolic compounds.^38,39^ Roasting at 170 °C altered the conformation of Ara h 2, increasing random coil content and reducing α-helix, but also enhanced IgE binding. However, roasting Ara h 1 and Ara h 6 increased IgE binding,^147^ while Ara h 2 and Ara h 3 showed no significant changes.^148^ These findings could be attributed to the dissimilar conformational structure of the allergens in the peanut protein.^148^ Boiling, autoclaving, and pressurized autoclaving significantly reduced allergenicity, with the greatest reduction observed when combined with enzymatic digestion, as confirmed by decreased wheal size on the skin and the absence of immunoreactive proteins.^43^ Apart from thermal treatments, apple polyphenols have been shown to reduce peanut allergenicity by binding to proteins. Treatment with polyphenols, particularly epicatechin, decreased IgE, IgG1, histamine, TNF-α, IL-4 levels, and clinical anaphylaxis. Epicatechin was the most effective, followed by catechin, chlorogenic acid, rutin, and phlorizin.^39^ Additionally, peanut protein conjugated with polyphenols like epigallocatechin-3-gallate and chlorogenic acid showed increased digestibility and decreased IgE-binding, while altering protein conformation and reducing allergenicity. *In vivo*, polyphenol-treated groups exhibited lower food allergy responses of serum IgE, IgG1, IgG, histamine, and Th2 cytokines (IL-4, IL-5, IL-13) levels and higher IFN-γ levels, indicating a reduced allergic response.^38^

Pistachio (*Pistacia vera*), one of the tree nuts, is attributed to allergenic reactions.^149,150^ Decreasing the allergenicity of pistachio allergens by thermal applications was a matter investigated by Cuadrado et al.^43^ Boiling (4.1 mm^2^), autoclaving (1.5 mm^2^), and pressurized heating (2.4 mm^2^) significantly reduced the wheal size on the skin compared to raw pistachio (>8 mm^2^). Autoclaving and pressurized heating decreased 7S globulin and LTP levels, but 2S albumin remained. Enzyme treatments combined with boiling, autoclaving, or pressurized heating caused protein fragmentation.^43^

The edible part of walnuts (*Juglans regia* L.) is rich in lipids, protein, and polyphenols.^151^ However, walnut protein may lead to allergy in an allergic person and may cause life-threatening effects such as shock and mortality.^152^ Covalent interactions between walnut proteins and polyphenols (extract from walnut pellicle) altered the proteins’ secondary and tertiary structure, promoting unfolding. Polyphenols also reduced the IgG-binding capacity of walnut proteins in a dose-dependent manner (26.90% min).^37^

### 3.4 Oilseeds

Sunflower (*Helianthus annuus*) proteins, while nutritionally valuable, contain allergenic components.^153^ Roasting sunflower seeds reduces some allergenic potential but can form reactive byproducts. Studies show that roasted seeds at 140 °C for 10 min can cause liver oxidative stress in rats.^154^ Case reports describe severe allergic reactions, including angioedema and respiratory distress, with sensitization to Hel a 3 and oleosins,^155^ and cross-reactivity between sunflower proteins and allergens from hazelnuts, egg whites, and oranges.^153^ Animal studies show that sensitized mice exposed to 2S-albumins, such as SESA2–1 and SESA20–2, exhibited strong Th2 cytokine responses and IgE reactivity, with cross-reactivity among 2S-albumins but not peanut allergens.^156^ Smaller proteins like Hel a 3 are potent allergens due to their stability and IgE-binding capacity, while oleosins evade standard diagnostic methods.^157^ Although sunflower proteins are less allergenic than soy or sesame, they can trigger severe reactions, including anaphylaxis, requiring careful food safety assessment.

Rapeseed (*Brassica napus L*.) contains allergenic proteins like cruciferin (11S globulin) and napin (2S albumin), with napins resisting heat and proteolysis, maintaining IgE-binding capacity. Bra o 3, a 9 kDa nsLTP, shares structural similarities with allergens from mustard and other cruciferous vegetables, causing cross-reactivity and severe reactions such as FDEIA.^158^ Fermentation with microorganisms like *Saccharomyces cerevisiae* and *Bacillus subtilis* resulted in partial degradation of cruciferin, potentially reducing allergenicity by breaking down stable epitopes.^36,159^ The decrease was linked to the breakdown of allergenic epitopes and structural modifications of proteins, reducing their recognition by IgE antibodies. Napins and nsLTPs remain the primary contributors to rapeseed allergenicity, necessitating further innovations to effectively reduce these risks.

Sesame (*Sesamum indicum*) is a significant allergenic food source is a major allergenic food with major allergens including 2S albumins (Ses i 1, Ses i 2), cupin family proteins (Ses i 3, Ses i 6, Ses i 7), and oleosins (Ses i 4, Ses i 5). Ses i 1 and Ses i 2 are the most stable and immunogenic due to their high disulfide bond content, which resists denaturation during processing.^160^ Processing methods, especially roasting at 180 °C for 5–30 min, alter sesame protein structure, increasing α-helix content and decreasing β-sheet content, reducing IgE-binding capacity, particularly for less stable allergens like oleosins. However, Ses i 1 and Ses i 2 retain their IgE reactivity due to their structural stability.^160,161^ Cold plasma treatment at 25–120 W reduces sesame protein allergenicity by 23% without compromising nutritional quality.^46^ Germination improves protein digestibility from 43.69% to 47.97% after 2 days and reduces allergenicity by altering protein structure, with SDS-PAGE showing breakdown of high-molecular-weight proteins (>40 kDa) into smaller peptides, and changes in secondary structure with reduction β-sheet content that disrupt allergenic epitopes.^162^ Glycated sesame proteins showed reduced IgE binding, histamine release, and β-hexosaminidase activity, indicating a lowered immune response.^93^ Additionally, cytokine levels associated with Th2-mediated allergic reactions, including IL-4, IL-5, and IL-13, were significantly suppressed in mice treated with glycated proteins, with glucose and galactose showing the greatest efficacy. Structural analyses revealed alterations in protein conformations, such as a reduction in α-helix and β-sheet content and decreased surface hydrophobicity, further contributing to the reduced allergenic potential by masking allergenic epitopes. These processing strategies collectively highlight their potential to mitigate sesame allergenicity while maintaining functional and nutritional quality.^93^ Furthermore, *in vitro* and *in viv*o studies confirm the strong allergenic potential of 2S albumins, especially Ses i 1. While roasting and enzymatic treatments reduce allergenicity for some proteins, highly stable allergens remain potent. Sesame allergy affects 0.1–0.9% of the population, with severe reactions, including anaphylaxis, reported.^163,164^ Roasting, HPP, and plasma treatment can reduce sesame allergenicity, but the stability of 2S albumins highlights the need for innovative methods to lower their immunogenicity while preserving sesame proteins’ functionality and nutrition

Hemp seeds (*Cannabis sativa L*.) contain allergenic proteins like 2S albumins, 7S vicilins, and 11S legumins, which are heat- and digestion-resistant with strong IgE-binding potential. Edestin and vicilin, particularly stable, cross-react with tree nut and oilseed allergens like hazelnut and sesame. In vivo studies show hemp proteins’ significant IgE-binding potential, with 2S albumins cross-reacting to peanut and tree nut allergens, and reduced IgE binding to hazelnut extracts when preincubated with hemp proteins, highlighting shared epitopes and allergenic risks.^165^ Studies show up to 30% inhibition of IgE binding between hemp and hazelnut proteins due to shared epitopes.^165^ Germination reduces allergenicity by altering protein profiles, increasing solubility from 10.3% to 21.8%, and enhancing foaming capacity, though its effect on IgE binding needs further validation.^166^ Enzymatic hydrolysis may reduce allergenic epitopes but can impair functional properties.^167^ Micellization extraction reduces allergenicity more than alkaline extraction, yielding higher albumin and sulfur-containing proteins while lowering allergenic proteins like Hsp70.^168^ Despite these advances, highly stable allergens like edestin remain challenging to mitigate, necessitating further research into optimizing allergenicity reduction while preserving functional and nutritional properties.

### 3.5 Insects

The investigation on edible insects is growing exponentially, particularly due to the increasing human population and due to varying concerns in the post-COVID era, such as the sustainable food supply chain. Currently, edible insects are classified into eight main orders, which are Blattodea (cockroaches and termites), Coleoptera (beetles), Diptera (flies), Hemiptera (bugs), Hymenoptera (bees, wasps, ants), Lepidoptera (butterflies, moths), Odonata (dragonflies), and Orthoptera (crickets, grasshoppers, locusts).^169^ Potential food-related allergens only include the brown garden snail (*Helix aspersa*) and the silk moth (*Bombyx mori*).^116^ Despite its crucial potential as a novel and sustainable protein source, insect consumption is one of the extreme neophobias for many people, not only for their nauseatingness but also for their critical allergenicity attributes.

In the past decade, there has been intense research to decipher the complete allergen potential of each insect. Recent research and review publications revealed that the mostly focused edible insects bear dozens of food allergen compounds. However, the major common allergens are tropomyosin and arginine kinase, which induce Th2-biased immune responses and reduce the activity of CD4+T regulatory cells.^15,170^ Cross-reactivity and anaphylaxis are the most common allergenic incidences for most edible insects, like crickets, locusts, and mealworms.^170,171^ Tropomyosin and arginine kinase have plenty of isoforms per species, besides other allergen compounds (enzymes, precursors) that overall allergen compound number, for instance, of silkworms reached over 30 as of today.^116,172^ Summarizing even briefly the allergen types of each species will not be suitable to stay in the scope of this present work; however, a comprehensive and detailed review study should be referred to for extensive information about allergen compounds for the most common edible insects.^172^

As a novel and alternative protein source for human consumption, processing is obligatory due to food safety and quality requirements. Furthermore, it is known that allergenicity risks of edible insects might be mitigated with processing due to the potential modifications of epitopes to reduce allergens IgE-binding abilities.^15^ The first studies in the literature claimed that most food processing methods were unable to mitigate allergen factors in edible insects.^169^ Since the major allergen, tropomyosin, is highly heat stable, hurdle/synergistic effects of multiple processing might be a better approach to combat insect allergens.^15^ Recent studies have managed to reduce the allergens for both IgE-binding abilities and cross-reactivities with other food allergens (Table 2). Nevertheless, broader studies are required to demonstrate the full potential of distinctive thermal/nonthermal processing techniques on edible insect allergens from varying species.

**Table 2:** Impact of Processing and Treatments on the Allergenicity of Food Proteins^a^

**Proteins**	**Treatments**	**Assays**	**Main results**	References
**LEGUMES**				
**White bean (*****Phaseolus vulgaris*****)**	Enzymatic hydrolysis: papain and alcalase	IgE-immunoblotting assay with allergic patients	→ImmunoreactivityPapain and Alcalase: Did not completely abolish immunoreactivity due to antinutritional factors.	(32)
**Mung bean (*****Vigna radiata*****L.)**	Enzymatic hydrolysis: papain, alcalase and flavorzyme	IgE-immunoblotting assay with allergic patients	↓ImmunoreactivityPapain hydrolysate: Some immunoreactivity remained, with new epitopes unmasked (26 kDa subunit of 8S globulin).	(31)
**Lupin (*****Lupinus albus*****)**	Fermentation via *Rhizopus. oligosporus*	Computational Allergenicity Prediction and Linear Epitope IdentificationMultiple Reaction Monitoring (MRM) Mass Spectrometry	↓Allergenicity: reduction in 96% of peptides; 83 decreased >50%.β-Conglutin: Significant N-terminal reductions linked to epitopes.α-Conglutin: 23 peptides reduced >50%.	(33)
			β-Conglutin: Significant N-terminal reductions linked to epitopes.	
			α-Conglutin: 23 peptides reduced >50%.	
			*δ-*Conglutin and nsLTP: Most decreased >40%.	
			γ-Conglutin: Minimal changes due to structural resistance.	
**Lupin (*****Lupinus luteus*****)**	Germination at 25 °C for 9 days	Mass spectrometry analysis	↓Allergenicity: Massive degradation of β-conglutin isoforms	(108)
**Lupin (*****Lupinus angustifolius*****)**	Roasting at surface temperatures of 98, 120, 140, 160, 175, 195, 220, and 242 °C	LC-MS/MS	↓Allergenicity	(109)
			α-conglutins: Stable up to 175–220 °C.	
			β-conglutins: Stable up to 175 °C, high abundance at 175 °C for β5 and β7.	
			δ-conglutins: Higher abundance at 140–175 °C for δ1 and δ3, reduced at 160–175 °C for δ2 and δ4.	
			γ-conglutins: Significant reduction at 120–140 °C.	
			Other proteins: Low stability, reduction at 120 °C, higher extraction at 98 °C	
**Chickpea (*****Cicer arietinum*****)**	Enzymatic hydrolysis: papain and alcalase	IgE-immunoblotting assay with allergic patients	↓Immunoreactivity	(32)
			Alcalase hydrolysis: No immunoreactivity detected, suggesting effective reduction of allergenicity.	
			Papain hydrolysis: Some immunoreactivity remained, with a new reactive protein (2S Albumin) detected, indicating unmasking of new epitopes.	
**Peas (*****Pisum sativum*****L.)**	Fermentation via *Lactobacillus plantarum* + Enzymatic hydrolysis: papain, Esperase, trypsin	ELISA via allergenic rabbit serum	↓Immunogenicity	(40)
			Fermentation reduced Pis s2 solubility, not proteolysis.	
			Pis s1 intensity decreased with fermentation and hydrolysis.	
			Combined treatments degraded proteins to smaller fractions.	
**Soybean (*****Glycine max*****)**	Autoclaving at 121 °C for 20 min	ELISA via patient serum	↓Allergenicity: boiling by 69.3%, autoclaving by 88.9%.	(211)
	Boiling at 100 °C for 20 min			
**CEREALS**				
**Wheat (*****Triticum aestivum*****)**	Sourdough fermentation	FODMAPs analysis and HPLC	↓α-Trypsin inhibitors level by 41%	(34)
	Sourdough fermentation	ELISA via allergenic rabbit serum	No synergistic effect	(26)
			Allergenicity initially increased, then decreased during fermentation.	
			Acidification enhanced protease activity, reducing allergenicity	
	Baking at 200 °C or steaming at 100 °C for 40 min		↓Gliadin specific inflammatory response	
			Baking reduced allergenicity more than steaming.	
			Fermentation increased allergenicity in steamed samples.	
			Steaming protected allergenic determinants via water binding.	
	Dough fermentation via *Pediococcus acidilactici XZ31* and *Saccharomyces cerevisiae JM1*	ELISA via patient serum or R5 Competitive	↓Amount of allergens across Caco-2 monolayer after the digestion of coculture fermentation	(35)
			↓Gluten translocation across the Caco-2 monolayer	
			Fermentation: ↓Albumin/globulin allergenicity and ↑R5 reactivity of gluten	
	Transamidation via microbial transglutaminase with/without l-lysine and/or GSH at 40 °C for 40 min and steaming for 20 min	R5 Competitive ELISA	↓Gliadin specific inflammatory response activity by 83%	(123)
			CD toxicity: 78% for transglutaminase alone, 56% transglutaminase with l-Lysine, 29% transglutaminase with l-lysine and GSH	
	Baking at 230 °C for 25 min	Digesta analysis via Quantitative Mass Spectrometry	↓Immunogenic peptide (33mer/P5) release by 20%	(79)
	High hydrostatic pressure treatment at 400 MPa for 20 min	ELISA	↓Gluten allergenicity by 72%	(45)
			No significant decrease in allergenicity at 500 MPa	
**Tartary buckwheat (*****Fagopyrum tataricum*****(L.) Gaertn.)**	Fermentation via *Lc*. *taiwanensis, W*. *cibaria*, or *P*. *pentosaceus*	ELISA	↓Up to 82 and 39.1% of IgE reactivity reduction for *P. pentosaceus* and *W. cibaria*	(212)
			*Lc. taiwanensis*: Showed unique allergen trends, with a decrease after 12–20 h.	
			12–20 h fermentation recommended for significant allergen reduction	
**Barley (*****Hordeum vulgare*****)**	Beer with *Trichoderma reesei-*expressed prolyl endopeptidase enzyme	R5 Competitive ELISA	↓Gluten quantity reduced to <20 ppm	(134)
**NUTS**				
**Cashew (*****Anacardium occidentale*****)**	Boiling at 100 °C for 60 min	Western blot and ELISA via patients’ sera, Skin prick test	↓Wheal size: Control > Boiling > Autoclaving > Pressured heating > Pressured heating + Enzymatic hydrolysis	(43)
	Autoclaving at 138 °C/256 kPa for 30 min			
	Pressured heating at 170 °C/7 bar for 120 s		↓Protein bands and IgE bounds except for boiling	
	Pressured heating + Enzymatic hydrolysis: Amano enzyme			
	Irradiation at 1, 3, 5, and 10 kGy	ELISA via patients sera	↓IgE binding capacity of Ana o 3	(44)
			↓Histamine, Cytokine levels	
**Hazelnut (*****Corylus avellana*****)**	Boiling at 100 °C for 60 min	Western blot and ELISA via patients sera, Skin prick test	Raw and boiled hazelnut had similar IgE binding profiles	(43)
	Autoclaving at 138 °C/256 kPa for 30 min		↓Wheal size	
	Pressured heating at 170 °C/7 bar for 120 s		↓Protein bands	
	Pressured heating + Enzymatic hydrolysis: Amano enzyme		↓Wheal size; up to 50% reduction postboiling and enzymatic treatment.	
			↓IgE bounds; Pressured heating + enzymatic hydrolysis almost eliminated IgE binding.	
	Autoclaving for 10 min	Western blot and ELISA via patients sera, Skin prick test	↓Wheal size and Allergens	(42)
	Prehydrating + Autoclaving: 134 °C/2 atm for 1 h		↓IgE reactivity: Drying after autoclaving has minimal effect	
	Prehydrating + autoclaving + drying overnight at 60 °C		Autoclaving reduces hazelnut allergenicity by degrading proteins.	
			Prehydrated/autoclaved hazelnuts show no reactive bands	
**Peanut (*****Arachis hypogaea*****L.)**	Roasting at 170 °C for 20 min	Western blot and ELISA and allergenicity in KU812	↓α-helix content higher ↑random coil	(147)
			↑IgE binding capacity	
			↑β-hexosaminidase	
			↑TNF-α monomers	
			↓Histamine	
	Roasting at 170 °C for 12 min	Western blot and ELISA	↑IgE binding capacity of Ara h 1 and Ara h 6	(148)
			↑Ability to elicit KU812 cell degranulation of Ara h 1 and Ara h 6	
			→IgE binding capacity of Ara h 2 and Ara h 3	
			→Ability to elicit KU812 cell degranulation of Ara h 2 and Ara h 3	
	Boiling at 100 °C for 60 min	Western blot and ELISA via patients sera, Skin prick test	↓Wheal size: Control > Boiling > Enzymatic hydrolysis > Pressured heating > Autoclaving	(27)
	Autoclaving reduces hazelnut allergenicity by degrading proteins.		↓Immunoreactive proteins, except for boiling	
	Pressured heating at 170 °C/7 bar for 120 s		↓% lgE binding inhibition: 63.4% for control, 62.7% for boiling, 27.8% for pressured heating, 22.1% for autoclaving, 29.3% for Pressured heating + Enzymatic hydrolysis	
	Pressured heating + Enzymatic hydrolysis: Amano food-grade proteases		→lgE % binding inhibition: 76.4% for enzymatic hydrolysis.	
	Interaction with polyphenols (picatechin, phlorizin, rutin, chlorogenic acid, catechin)	Specific IgE and IgG1 antibodies of mouse via ELISA	↓IgE and IgG1 levels	(39)
			↓Clinical anaphylaxis	
			↓Histamine	
			↓TNF-α levels	
			↓IL-4 levels	
	Conjugation with polyphenols (epigallocatechin-3-gallate and chlorogenic acid)	Western blot and ELISA and allergenicity in KU812	↓Allergenicity	(38)
			↑Digestibility	
			↓IgE-binding capacity	
			↓lgE, IgG1, IgG,	
			↓Histamine	
			↓Th2 cytokines (IL-4, IL-5, IL-13)	
			↑IFN-γ l	
**Pictachio (*****Pistacia vera*****)**	Boiling at 100 °C for 60 min	Western blot and ELISA via patients sera, Skin prick test	↓Wheal size: Control > Boiling > Pressured heating > Autoclaving > Pressured heating + Enzymatic hydrolysis	(43)
	Autoclaving at 138 °C/256 kPa for 30 min			
	Pressured heating at 170 °C/7 bar for 120 s		↓Protein bands and IgE binding except boiling	
	Pressured heating+Enzymatic hydrolysis (Amano enzyme, 1 mg/mL)			
**Walnut (*****Juglans regia*****L.)**	Interaction with phenolic extracts from walnut pellicle	ELISA via rabbit serum	Change in secondary and tertiary structure	(37)
			↑Unfolding of the protein	
			↓IgE-binding capacity	
**OILSEEDS**				
**Sunflower (*****Helianthus annuus*****)**	Roasting at 140 °C for 10 min	*In vivo* Wistar rats	↓Allergens (not specified)	(154)
			↑Reactive byproducts	
			↑Liver oxidative stress and structural damage	
**Rapeseed (*****Brassica napus*****)**	Toasting and oil extraction	Mass spectrometry analysis	→Napin amount	(208)
			↓30% in Cruciferin amount	
**Sesame (*****Sesamum indicum*****)**	Roasting at 180 °C for 5–30 min	Western blot and ELISA via patients’ sera	↑α-helix content from 11% to 25% ↓β-sheet content from 36% to 17% after 30 min roasting	(160)
			↓up to a 50% in IgE-binding capacity of oleosins (Ses i 4 and Ses i 5)	
			→IgE-binding capacity of Ses i 1 and Ses i 2	
	Cold plasma at 25, 60, and 120 W	ELISA	↓Allergenicity by 1.34, 5.8, and 2.9 decrease in binding at 25, 60, and 120, respectively.	(46)
	Glycation at 100 °C for 30 min with different saccharides: glucose, galactose, lactose, and sucrose	*In vivo* Wistar rats	↓IgE-binding capacity: 4–52% reduction	(93)
			↓Histamine, Th2 cytokine Levels, and β-Hexosaminidase levels	
			↓Th2 cytokine production	
			No significant difference on Th1 Cytokine (IFN-γ) among groups	
**Hemp seed (*****Cannabis sativa*****)**	Micellisation extraction and freeze-drying	Label-free quantitative proteomic analysis using nanoLC-QTOF-MS	↓Allergenic potential compared to alkaline extraction	(168)
			↓Allergenic proteins like Hsp70	
			↓Allergenic proteins by freeze-drying compared to spray drying and nondry	
**INSECTS**				
**Mealworm (*****Tenebrio molitor*****)*****(Zophobas atratus) (Alphitobius diaperinus)***	Boiling, frying and lyophilizing to test their cross-reactivity for shrimp and house dust mite allergies	Dot Blot and SDS-PAGE and Immunoblotting	↓Cross-allergenicity for tropomyosin, α-amylase, hexamerin 1B precursor and muscle myosin	(213)
**Mealworm (*****Tenebrio molitor*****) Buffalo worm*****(Alphitobius diaperinus)*****Silkworm*****(Bombyx mori)*****Cricket*****(Gryllus campestris)***	Boiling and frying the insects to test their cross-reactivity for shrimp and house dust mite allergies	Dot Blot and Western Blot	↓Cross-allergenicity dependent on protein, species and treatment type, quite various but promising	(30)
**Cricket*****(Gryllus campestris)***	Microwave heating at 600 W and Enzyme treatment: Alcalase	Western blot and ELISA via patients’ sera	↓IgE and IgG reactivity significantly for 31 epitope regions	(28)
**Silkworm pupae*****(Bombyx mori)***	Heating: 20–120 °C for 20 min	Western blot and ELISA via patients’ sera and allergenicity in KU812	↓Allergenicity decreased significantly at temperatures >80 °C, with 25–33 kDa allergens remaining heat-resistant at 100 °C after 30 min	(214)
	Heating: at 60 °C, 80 and 100 °C for 5–30 min		↓Histamine release	
	Pepsin and Trypsin Digestion		Acid and alkaline: Acidic conditions (pH 1.0–3.0) degraded	
	Acid and alkaline treatments for 16 h		Digestion: Pepsin more effective	
**Cricket (*****Acheta domesticus*****)**	Roasting and seasoning	Dot Blot, Prick to Prick Testing, SDS-PAGE and Immunoblotting, IgE Inhibition Assay, LC-MS	↓Allergenicity for 45 and >97 kDa to only 45 kDa	(10)
**Superworm (*****Zophobas morio*****F.)**	Blanching for 5 min and Ultrasound treatment at 100 W for 30 min	ELISA	↑Allergenicity for blanching (2- or 3-fold)	(215)
			↓Allergenicity only for ultrasound treatment at 50 °C	
**Honeybees (*****Apis mellifera*****)**	Nanomaterial treatment (magnetic nanocomposite with photo/chemical synergistic capability)	Western Blot	↓IgE binding level of Phospholipase A2 (PLA2) by around 50%	(216)
**MACRO-ALGAE**				
***Ulva*****sp**.	Pulsed Electric Fields (PEF) at 12 or 26 kV, osmotic shock, thermochemical extraction	*In silico* allergenic risk	↓Troponin C with PEF	(20)
			→Nutritional quality	
Altered secondary structures				

a nsLTP: nort specified Lipid Transfer Protein.

### 3.6 Macroalgae and Microalgae

The increasing use of algae-derived ingredients in the food industry has raised concerns about their potential allergenicity. Algae, including both macroalgae (seaweed) and microalgae, contain proteins and polysaccharides that can trigger allergic reactions in susceptible individuals.^173^ Scientific studies have identified macroalgae, particularly red and brown seaweeds, as potential sources of food allergens. Among red seaweeds, species such as *Chondrus crispus*, *Palmaria palmata*, and *Porphyra* (commonly known as *Nori*) have been associated with IgE-mediated allergic reactions, leading to symptoms such as urticaria, angioedema, and respiratory distress.^174^

One of the most widely used polysaccharides derived from red seaweed is carrageenan, a common food additive with emulsifying and gelling properties. However, carrageenan has been implicated in allergic responses, including IgE-mediated anaphylaxis and hypersensitivity reactions.^175,176^ The presence of the α-gal epitope in carrageenan has also been suggested as a contributing factor to allergic cross-reactions with mammalian meat allergens, further increasing the risk for sensitive individuals.^177^

Brown seaweeds, such as *Saccharina latissima*, have been reported to contain proteins structurally similar to known crustacean allergens, including tropomyosin, arginine kinase, and myosin light chain. This structural similarity raises concerns about cross-reactive allergic responses among individuals allergic to shellfish.^178^ Additionally, macroalgae cultivated in marine environments may become contaminated with shellfish allergens, further complicating allergenic risk assessments.^179,180^*In vivo* studies demonstrate the allergenic potential and mitigation strategies for algae-derived proteins. Human sera studies also confirmed IgE cross-reactivity between algal proteins and seafood allergens, emphasizing the need for precautionary labeling.^19^ While these findings suggest that macroalgae may contribute to food allergies, the exact prevalence and mechanisms remain poorly understood, warranting further investigation.

Microalgae, particularly *Spirulina* (*Arthrospira*) and *Chlorella*, have been more frequently associated with allergic reactions, including anaphylaxis.^181,182^ The most studied allergenic protein in *Spirulina* is the C-Phycocyanin Beta Subunit (15–35 kDa), which has been implicated in severe allergic responses, including anaphylaxis.^183^ Other potential allergenic proteins in *Spirulina* include thioredoxin (13–14 kDa), superoxide dismutase (20–25 kDa), and glyceraldehyde-3-phosphate dehydrogenase (35–40 kDa), identified based on their structural similarity to known food allergens.^183^

Similarly, *Chlorella* contains IgE-binding proteins ranging from 13 to 72 kDa, with Tiberg et al.^184^ demonstrating that *Chlorella homosphaera* harbors multiple allergenic protein fractions. Yim et al.^185^ further reported cases of acute tubulointerstitial nephritis associated with *Chlorella*-based supplements, suggesting that algal proteins may not only trigger IgE-mediated allergies but also other immune-related hypersensitivity reactions.

Processing methods significantly influence the stability and detectability of allergenic proteins in algae. Bianco et al.^186^ investigated the impact of thermal processing on *Spirulina*-derived allergens and found that while some proteins degraded during baking, others, particularly from C-Phycocyanin Beta Subunit, remained stable in processed foods such as biscuits, pasta, fruit juice, and crackers. However, in high-temperature processed products like biscuits, partial degradation of allergenic proteins was observed, suggesting that processing conditions affect allergen stability to varying degrees. The study assessed the allergenic potential of proteins extracted from *Ulva* sp. macroalgae using osmotic shock, mechanical pressing, and pulsed electric fields.^20^ Pulsed electric field applied extracts contained superoxide dismutase and troponin C, while thermochemical extraction produced aldolase A and thioredoxin h, potential allergens. Pulsed electric fields selectively limited troponin C release, potentially reducing allergenic risks by disrupting cell membranes and altering protein structures, modifying allergenic epitopes.^20^

Heat treatment partially denatures proteins, reducing IgE reactivity by up to 30% for less stable proteins, but highly stable ones like phycobiliproteins and tropomyosin remain unaffected.^173^ Roasting macroalgae alters protein profiles, reducing allergenicity for some, while robust allergens retain reactivity.^187^ Enzymatic hydrolysis effectively degrades allergenic epitopes, reducing IgE-binding potential but can impair functional properties if excessively applied.^78^

Studies using proteomics and in silico homology analysis have confirmed that certain microalgal proteins exhibit cross-reactivity with known food allergens, potentially triggering IgE-mediated responses. Gregory et al.^19^ demonstrated that recombinant Ara h 1 and Ara h 2 allergens produced in *Chlamydomonas reinhardtii* had reduced IgE binding compared to native peanut allergens, suggesting their potential as safer alternatives in immunotherapy. In contrast, *Chlorella* proteins such as calmodulin and fructose-bisphosphate aldolase have shown homology with crustacean allergens, raising concerns about sensitization risks.^188^

Despite the increasing consumption of algae-based foods, labeling regulations for algal allergens remain insufficient in both the EU and the US, where only major allergens such as peanuts, milk, and shellfish are required to be declared.^189^ Given the growing market for algae-derived proteins and bioactives, more research is needed to determine the prevalence of algal allergies in the general population and to biochemically characterize potential allergens. Additionally, measures should be taken to prevent cross-contamination between algae and seafood allergens in food production systems.

Interestingly, some algae-derived bioactive compounds have demonstrated strong antiallergic properties. For instance, murine models fed with brown seaweed showed reduced allergic inflammation through the suppression of T-helper 2 cytokines (IL-4, IL-5, IL-13) and modulation of adaptive immunity.^187^ Similarly, immunotherapy using algal-produced proteins reduced peanut-induced anaphylaxis in mice by decreasing IgE-binding and inflammatory responses.^190^ Sugiura et al.^190^ and Bae et al.^191^ reported that *Chlorella vulgaris* water extract suppresses histamine release and modulates Th1/Th2 balance, reducing IgE overproduction and allergic inflammation. Similarly, fucoidans from brown seaweeds (*Undaria pinnatifida, Fucus vesiculosus, Laminaria japonica*) have shown antiallergic effects by inhibiting mast cell degranulation and reducing histamine release.^192^ Phlorotannins from brown algae (*Ecklonia cava, Eisenia bicyclis*) have been found to suppress IL-4 and IL-13 expression, block IgE-mediated responses, and inhibit mast cell activation.^193^

While algae-based foods offer a promising alternative in sustainable nutrition, their allergenic risks must be carefully evaluated. Future studies should focus on identifying allergenic proteins, assessing their immunogenicity, and conducting large-scale epidemiological studies to better understand their impact on public health. Additionally, food safety frameworks should be revised to include algal allergens in labeling regulations, ensuring better consumer protection.

### 3.7 Mycoproteins

Mycoproteins, primarily derived from *Fusarium venenatum*, have gained significant attention as sustainable protein alternatives, yet their allergenic potential remains a concern due to cross-reactivity with fungal allergens and mold proteins.^194^ While generally considered safe, certain components within mycoproteins may trigger immune responses in susceptible individuals. High molecular weight proteins present in mycoproteins can be recognized by the human immune system, further contributing to allergenic potential.^13^ Furthermore, specific fungal proteins, including enolase and triose-phosphate isomerase, have been identified as cross-reactive allergens, meaning that individuals sensitized to airborne fungi or other fungal allergens may experience adverse reactions upon mycoprotein consumption.^195^

While generally regarded as low in allergenicity, severe allergic reactions have been reported in mold-sensitive individuals, with cases of anaphylaxis, respiratory distress, and gastrointestinal symptoms documented after mycoprotein consumption.^195^ For example, a 16-year-old with mold allergies experienced anaphylaxis after consuming Quorn patties, attributed to cross-reactivity between fungal proteins and airborne mold allergens.^13^ However, controlled studies involving 30 volunteers who underwent skin-prick tests after consuming *F. venenatum* mycoprotein showed no signs of allergic sensitivity, reinforcing the notion that severe allergic responses are rare and mostly occur in predisposed individuals.^13^ In controlled studies, skin-prick tests on 30 volunteers consuming *F. venenatum* mycoprotein showed no signs of allergic sensitivity, supporting its safety for the general population.^196^

Fungal proteins in mycoprotein products can exhibit cross-reactivity with common mold allergens, leading to IgE-mediated hypersensitivity reactions. Hoff et al.^197^ confirmed allergic cross-reactivity between mycoproteins and mold allergens, identifying the 60S acidic ribosomal protein P2 as a primary allergenic component in *F. venenatum*. This protein was found to be highly homologous to *Fusarium culmorum* allergens (Fus c 1) and also exhibited cross-reactivity with *Cladosporium herbarum*, *Aspergillus fumigatus*, and *Alternaria alternata*. Their clinical case study of a mold-sensitive patient experiencing an immediate-type hypersensitivity reaction after consuming Quorn suggests that individuals with mold allergies or asthma are at a higher risk of developing allergic reactions to mycoproteins. Xing et al.^195^ further explored fungus food allergy syndrome (FFAS), a phenomenon in which sensitization to airborne fungal spores (e.g., *Aspergillus*, *Cladosporium*, and *Alternaria*) results in adverse immune responses to mycoprotein ingestion. The study highlighted that fungal allergen contain conserved protein domains, which can trigger oral allergy syndrome (OAS), gastrointestinal distress, respiratory issues, or even systemic anaphylaxis in mold-sensitive individuals.

Processing techniques play a crucial role in reducing mycoprotein allergenicity by altering protein structures and mitigating immune recognition. One of the most effective approaches is submerged fermentation (SmF), which has been demonstrated to enhance digestibility and reduce allergen content in microbial proteins. For instance, studies on fermented soy meal processed via SmF reported a 37.97% increase in digestibility and a complete elimination of detectable soy allergens, showcasing its potential for mycoprotein allergen mitigation.^198^ Thermal processing is another critical factor in allergen reduction, as heat denaturation disrupts IgE-binding epitopes, thereby reducing allergenicity. However, excessive thermal processing may degrade essential amino acids and compromise the overall nutritional quality of mycoproteins. In the case of Quorn products, thermal treatments combined with binder incorporation (e.g., egg albumen) have been found to alter the allergenic profile, necessitating careful optimization to balance allergen reduction and product functionality.^194^

Furey et al.^199^ conducted a comprehensive allergenicity evaluation of *Fusarium* str. *flavolapis* (Fy Protein), a novel mycoprotein, following Codex Alimentarius guidelines. Their study incorporated bioinformatics screening, in vitro digestibility tests, and allergenic cross-reactivity assessments, concluding that Fy Protein was moderately digestible, lacked high-risk allergens, and did not trigger significant immune responses in controlled studies. However, they cautioned that individuals with pre-existing *Fusarium* mold allergies could still experience cross-reactivity, emphasizing the need for further clinical testing. Bartholomai et al.^200^ expanded on this by using advanced computational allergenicity prediction tools (e.g., AlgPred 2.0, AllerTOP, and AllergenOnline) to screen mycoproteins for IgE-binding motifs and conserved allergenic domains. Their findings revealed that certain *Fusarium* proteins contain IgE-binding regions, indicating a theoretical risk of allergenicity. However, they noted that bioinformatics models often overestimate allergenic potential, and actual immune responses should be validated through *in vivo* and clinical studies.

Despite the identified risks, clinical studies have reinforced the overall safety of mycoprotein-based foods. Regulatory agencies, including the EFSA and FDA, recognize Quorn as safe for consumption, with reported allergy cases remaining exceptionally rare relative to total servings consumed. Mycoproteins present a promising sustainable protein alternative, but their allergenic potential remains a concern for mold-sensitive individuals due to cross-reactivity with fungal allergens. While most individuals tolerate mycoproteins without issues, documented cases of severe allergic reactions, including anaphylaxis, highlight the need for continued allergen screening and processing optimization. Advances in fermentation technology, bioinformatics-based risk assessments, and clinical allergen evaluation can further ensure the safety and widespread acceptance of mycoproteins as a viable alternative protein source for future food systems.

## 4 Future Trends and Conclusions

Emerging efforts, such as the ImpARAS project and advancements in machine learning models, are focusing on improving allergenicity prediction by enhancing the understanding of Adverse Outcome Pathways (AOPs) and implementing innovative testing methods. Despite these advancements, there remains a significant lack of systematic global initiatives for screening allergen risks and prevalence. This gap underscores the urgent need for continuous monitoring and comprehensive risk management strategies for novel proteins.

Studies on allergenic proteins in cereals and nuts highlight the complexity of food allergenicity, emphasizing the necessity of tailored mitigation approaches. While conventional methods such as thermal and enzymatic treatments are still widely used, novel strategies like phenolic compound interactions and nonthermal processing methods show potential for reducing allergenic risks. However, further research—especially in vivo studies and human clinical trials—is essential to confirm the efficacy and safety of these approaches. A deeper understanding of allergen-protein interactions and their structural dynamics is critical for developing hypoallergenic food products, ultimately enhancing the quality of life for individuals with food allergies.

Effective allergen risk management in food production is crucial to prevent life-threatening reactions, relying on accurate labeling, traceability, and adherence to international guidelines such as those established by Codex Alimentarius. Nevertheless, challenges persist due to inconsistent global allergen lists, unclear precautionary labeling, and gaps in the implementation of best practices. Undeclared allergens remain a leading cause of food recalls, highlighting the need for improved education, regulation, and monitoring systems. As global demand for sustainable and alternative protein sources grows, addressing the allergenicity of these novel ingredients becomes increasingly important for ensuring food safety and consumer acceptance.

Future trends in addressing allergenicity in alternative proteins are promising. Emerging technologies such as enzymatic hydrolysis, fermentation, glycation, and high-pressure processing show potential in reducing allergenic risks, with future research likely adapting these methods to specific protein sources. Advances in genetic engineering, including CRISPR, may enable the removal or modification of allergenic epitopes, leading to hypoallergenic protein variants. Omics technologies, such as proteomics, genomics, and metabolomics, are becoming indispensable for identifying and characterizing allergenic proteins, while bioinformatics tools enhance the prediction of allergenicity and cross-reactivity.

Unconventional protein sources, such as microalgae, fungi, and insect proteins, are increasingly studied, with a focus on understanding their allergenic potential and developing mitigation strategies. Hybrid proteins, blending sources like plant and insect proteins, may dilute allergens while maintaining functional properties. Immunotherapy research, including oral immunotherapy and peptide-based vaccines tailored to alternative protein allergies, is expanding. Additionally, advancements in personalized nutrition may lead to individualized diets addressing specific allergenic sensitivities to alternative proteins.

Despite these promising trends, several challenges remain. Limited data exist on the allergenic properties of many emerging proteins, including macro- and microalgae, mycoproteins, and insect-derived proteins. Cross-reactivity studies, particularly between insect proteins and shellfish allergens, require further exploration. The lack of globally accepted methods for assessing the allergenicity of alternative proteins complicates the comparability of research findings. Regulatory gaps also persist, with current frameworks often focused on conventional allergens, leaving novel protein allergens under-addressed. Comprehensive allergen databases that include alternative proteins are urgently needed to guide food safety assessments.

Mitigation techniques, such as enzymatic hydrolysis, may reduce allergenicity but could compromise protein functionality, including solubility or emulsifying capacity. Reports of severe allergic reactions linked to alternative proteins may undermine consumer confidence in these products, and advanced mitigation techniques might increase production costs, affecting the competitiveness of alternative proteins. Ethical and sustainability considerations also arise, as some allergen-reduction techniques may conflict with sustainability goals or ethical standards in food production. Finally, the scarcity of in vivo studies and human clinical trials evaluating the allergenicity of alternative proteins presents significant challenges for regulatory approval and consumer confidence.

In conclusion, ensuring the safe integration of alternative proteins into global food systems requires a multifaceted approach to address allergenicity. Future research should combine food science, immunology, and regulatory perspectives to overcome existing challenges. Bridging these gaps will enable the development of hypoallergenic, sustainable, and functional alternative protein products, supporting both food safety and consumer acceptance. Additionally, nutritional interventions, including hydrolyzed proteins, vitamins, probiotics, and fatty acids, hold potential for reducing food allergies and inducing oral tolerance. However, overcoming challenges like individual differences and unknown mechanisms will be crucial to developing permanent solutions. Furthermore, innovative strategies, such as creating covalently bound protein-phenolic complexes, offer a promising approach for mitigating food allergies. Extensive research, including both *in vitro* and *in vivo* studies, will be necessary to evaluate the efficacy of these strategies in reducing the allergenicity of legumes and other novel protein sources.

## Abbreviations

ELISA: Enzyme-Linked Immunosorbent Assay
FDEIA: Food-dependent exercise-induced anaphylaxis
IgA: Immunoglobulin A
IgE: Immunoglobulin E
IgG1: Immunoglobulin G1
IL: Interleukin
kDa: Kilodalton
LTP: Lipid Transfer Protein
mLNs: Mesenteric lymph nodes
PR-10: Pathogenesis-related protein 10
SDS: Sulfate Detergent Solution
SDS-PAGE: Sodium Dodecyl Sulfate Polyacrylamide Gel Electrophoresis
Th2: T-helper cell type 2
Tfh13: T follicular helper cells
TNF-α: Tumor Necrosis Factor Alpha
Treg: Regulatory T cells
WHO/IUIS: World Health Organization/International Union of Immunological Societies
